# Reassessing combined exercise–tDCS strategies in chronic pain

**DOI:** 10.1016/j.lana.2026.101379

**Published:** 2026-01-29

**Authors:** Stéphane Perrey

**Affiliations:** EuroMov Digital Health in Motion, University Montpellier, IMT Mines Ales, France

The factorial trial by Fregni et al.^1^ addressed two long-standing questions in chronic pain research that have significant implications for clinical guidelines and health policy. The first question is whether the dose of exercise–operationalised here as an aerobic versus non-aerobic stimulus–produces differential neurophysiological and clinical effects. The second question is whether combining exercise with motor-cortex neuromodulation meaningfully enhances outcomes. The findings challenge several assumptions that continue to influence recommendations for fibromyalgia.

First, the lack of clinically significant differences between aerobic and non-aerobic exercise highlights the need to reconsider exercise dosage beyond cardiometabolic thresholds.^2^ Although aerobic exercise is often favoured in guidelines, low-intensity, sub-aerobic activity, when delivered in a supervised manner, can achieve comparable improvements in pain, fatigue, sleep and quality of life.^3^ From a health policy perspective, this is significant: non-aerobic exercise is more accessible and safer for patients with low tolerance or comorbidities, and it can be implemented more easily across healthcare systems. The findings support a shift towards individualised, preference-concordant exercise prescriptions rather than intensity-driven mandates.^4^

Second, motor-cortex tDCS demonstrated a clear mechanistic effect by enhancing conditioned pain modulation, independent of exercise modality. However, this neurophysiological gain did not translate into superior short-term clinical outcomes. This dissociation highlights a critical issue in translational pain research: improvements in surrogate mechanistic markers do not necessarily confer additive clinical benefit within pragmatic timeframes.^5^ For policy health decisions, these results argue against routine implementation of combined tDCS-exercise protocols solely to enhance symptom outcomes, at least in short-term treatment models.

These findings support reconsideration of guideline priorities towards interventions that are aligned with patient tolerance rather than intensity-based norms. Non-aerobic exercise offers a feasible first-line option with equity and implementation advantages, while adjunctive neuromodulation should be reserved for situations where additional clinical benefit is clearly demonstrated.

## Contributors

SP wrote and edited the manuscript.

## Data sharing statement

NA.

## Declaration of interests

I declare no competing interests.
